# SOCIAL SUPPORT, RESIDENTIAL SEGREGATION, AND HEALTH OF OLDER CHINESE AMERICANS

**DOI:** 10.1093/geroni/igad104.1411

**Published:** 2023-12-21

**Authors:** Yiming Ma, Yanping Jiang, Stephanie Bergren, Fengyan Tang

**Affiliations:** Rutgers University, New Brunswick, New Jersey, United States; Rutgers University, New Brunswick, New Jersey, United States; Rutgers, the State University of New Jersey, New Brunswick, New Jersey, United States; University of Pittsburgh, Pittsburgh, Pennsylvania, United States

## Abstract

The limited but increasing body of research on residential segregation among U.S. older Asian Americans shows inconsistent findings on the health impact of residential segregation. These mixed findings suggest that the relationship between residential segregation and health may depend on some individual characteristics. This study aimed to investigate the moderating effect of perceived social support on the relationship between residential segregation and the two health outcomes: depressive symptoms and cognitive function among older Chinese Americans. Data were drawn from 3,097 older Chinese immigrants from the PINE Study in 2011-2013. Residential segregation was measured by the Index of Concentrations at the Extremes (ICE) at census tract level, which captured the concentration of Chinese-only speakers and English-only speakers at extreme. The social support measurement was the perceptions of social support provided by spouse, family, and friends. Depressive symptoms were measured by Patient Health Questionnaire (PHQ-9). Cognitive function was assessed by Mini-Mental State Examination, executive function, working memory, and episodic memory measures. Results showed a positive and significant moderating impact of social support on the relationship between ICE and depressive symptoms (β=0.086, p=0.034). Specifically, living in neighborhoods segregated towards Chinese-speaking was associated with fewer depressive symptoms at high, but not low levels of perceived social support. There were no moderation effects of perceived social support on the relationship between ICE and cognitive function. These results indicate the protective effect of living in neighborhoods segregated towards Chinese-speaking on depression, but only when older Chinese immigrants have high levels of perceived social support.

